# A Ca^2+^ cycling defect connects insulin resistance and heart failure

**DOI:** 10.1093/lifemeta/loac014

**Published:** 2022-08-04

**Authors:** Qian Shi, Duane D Hall, Long-Sheng Song

**Affiliations:** Division of Cardiovascular Medicine, Department of Internal Medicine, Abboud Cardiovascular Research Center, Carver College of Medicine, University of Iowa, Iowa City, IA 52242, USA; Division of Cardiovascular Medicine, Department of Internal Medicine, Abboud Cardiovascular Research Center, Carver College of Medicine, University of Iowa, Iowa City, IA 52242, USA; Division of Cardiovascular Medicine, Department of Internal Medicine, Abboud Cardiovascular Research Center, Carver College of Medicine, University of Iowa, Iowa City, IA 52242, USA

**In a recent study published in *Life Metabolism*, Quan *et al*. reported that intracellular Ca** ^**2+**^ **dysregulation in cardiomyocyte can be both a cause and an effect of cardiac insulin resistance that ultimately leads to diabetic cardiomyopathy.**

Numerous epidemiologic studies across several decades indicate that insulin-resistant states such as diabetes and obesity are risk factors for congestive heart failure independent of common cardiovascular disease factors such as hypertension and coronary artery diseases [1, 2]. On the other hand, there is evidence that heart failure itself causes the onset and development of insulin resistance [3]. Restoring normal cardiac output in advanced heart failure patients with left ventricular assistance devices improves insulin sensitivity and cardiac metabolism [4]. These observations suggest a bidirectional link exists between insulin resistance and cardiac contractile dysfunction. One common feature of both insulin resistance and heart failure could be abnormal intracellular Ca^2+^ homeostasis. Ca^2+^ handling dysfunction is a well-defined hallmark of heart failure [5], and it has long been hypothesized that elevations of cytosolic free Ca^2+^ in insulin-targeted cells may lead to the development of insulin resistance [6]. However, the mechanism by which a Ca^2+^ cycling defect could determine insulin resistance has not been pursued. Furthermore, it remains unclear whether the Ca^2+^ handling processes that are known to be dysfunctional in heart failure are the same as those that contribute to insulin resistance.

In a recent study published in *Life Metabolism* [7], Quan *et al*. provided compelling evidence that Ca^2+^ dysregulation can be both a cause and an effect of cardiac insulin resistance that ultimately leads to diabetic cardiomyopathy (DCM). The researchers fed mice up to 8 months with a diet enriched in both sugar and fat similar to the western diet (WD) human consume daily. WD-fed mice became obese after 1 month and started to show systemic insulin resistance as well as decreased heart contractile function after 4 months. Subsequently, the researchers analyzed the Ca^2+^ handling properties of cardiomyocytes isolated from these diabetic mice. Results showed impaired cytosolic Ca^2+^ reuptake rate into the sarcoplasmic reticula (SR) that was accompanied with decreased phosphorylation of SR/ER-Ca^2+^ ATPase, SERCA2a, at Thr^484^. These results confirmed that a WD leads to cardiac insulin resistance and the development of DCM in mice.

The researchers further investigated the potential role of SERCA2a-Thr^484^ phosphorylation in the development of DCM by generating a mouse model carrying a phospho-incompetent mutation of SERCA2a (Thr^484^Ala), mimicking its pathogenic state in DCM. By using this novel mouse model, the researchers demonstrated that the SERCA2a-Thr^484^Ala mutation impairs Ca^2+^ homeostasis, i.e. a significant decrease in SR Ca^2+^ release and a marked prolongation in SR Ca^2+^ reuptake rate, in cardiomyocytes. Remarkably, the researchers also observed that, although whole body glucose tolerance is not impaired in mutant mice, mutant cardiomyocytes are less responsive to insulin, indicating they are insulin-resistant. This critical finding reveals a potential bidirectional link between Ca^2+^ handling and cardiac insulin signaling mediated by SERCA2a function. Previously, the same group of researchers used genetic PKB/AKT or SPEG inactivation mouse models to nicely demonstrate that cardiac function is regulated through an insulin-PKB/AKT-SPEG-SERCA2a-Thr^484^ axis [8, 9]. Here, the new Thr^484^Ala mouse model suggests that SERCA2a is not only a target of the insulin-AKT-SPEG pathway, but also a potential regulator of the insulin pathway during the early phase of DCM development [7]. Following this finding, the researchers examined the abundance of key molecules involved in insulin signaling pathway and detected decreased protein levels of insulin receptor β subunit (IRβ) in cardiac tissue from WD-fed mice and in SERCA2a-Thr^484^Ala animals. Diminished IRβ protein levels were also observed after pharmaceutical depletion of SR Ca^2+^ content, suggesting that insufficient SR Ca^2+^ correlates with decreased IRβ protein levels. Mechanistically, the researchers focused on a Ca^2+^-dependent protease enzyme called Furin that cleaves IR precursor proteins to produce functional IRα and IRβ. Decreased SR Ca^2+^ content due to either genetical or pharmaceutical inhibition of SERCA2a results in lysosomal degradation of Furin, which ultimately leads to less activated IRβ protein presented to the plasma membranes. Of interest, the researchers showed that SERCA2a-Thr^484^Ala mutant mice have significantly decreased cardiac contractile function compared with WT mice beginning at a very young age (2-month-old) that was independent of gender. Interestingly, long-term WD feeding induced contractile dysfunction to a similar level in both WT and mutant animals, suggesting SERCA2a dysfunction-mediated insulin resistance occurs during the early phase of DCM with later pathological changes attributable to yet to be identified factors. Importantly, WD feeding of SERCA2a-Thr^484^Ala mice led to a similar degree of obesity and systemic insulin resistance as that of WT mice. These results clearly indicate that SERCA2a-Thr^484^ phosphorylation-mediated Ca^2+^ reuptake is important for both cardiac function under normal conditions as well as in the early pathogenesis of DCM induced by the WD.

**Figure 1:**
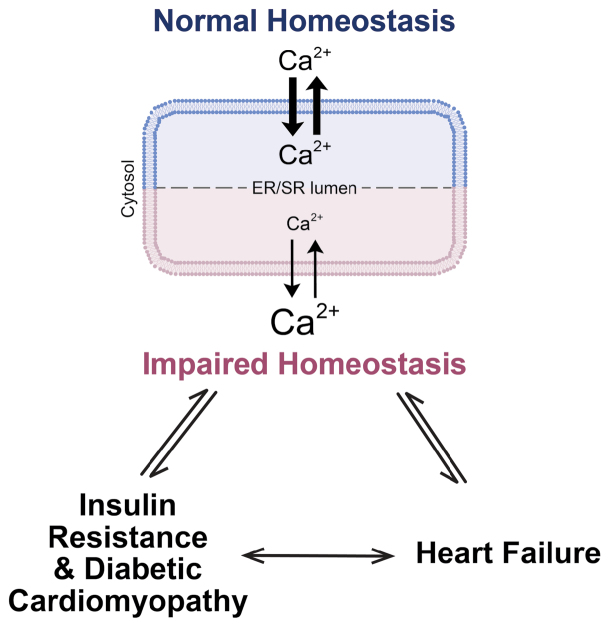
Diagram displaying the mechanistic relationship between Ca^2+^, insulin resistance, and heart failure.

To summarize, this study provides convincing evidence of a bidirectional relationship between Ca^2+^ dysregulation and cardiac insulin resistance which together contribute to the early pathogenesis of DCM. Insufficient Ca^2+^ reuptake into the SR not only negatively influences contractile function, but also impairs the processing of insulin receptor precursors and results in cardiac insulin resistance (Fig. 1). Besides phosphorylation at Thr^484^, the activity of SERCA can be modulated via other posttranslational modifications and regulators including sumoylation at a nearby residue [10], accessory proteins (phospholamban) or micropeptides (DWORF [11]). Changes in these regulatory factors might also contribute to the pathogenesis of insulin resistance and DCM and are worth testing in the future.

While impaired phosphorylation of SERCA2a-Thr^484^ underlies the early phase of DCM, no early onset of a middle phase was observed in this mutant mouse, suggesting that other factors may also coexist and are involved in the development of DCM. In addition, Ca^2+^ dysregulation is required but not sufficient to boost DCM progression. Therefore, identifying factors in pathogenesis of DCM in all phases is of great importance for optimizing therapeutic targets in the future. Other open questions include whether the insulin-PKB/AKT-SPEG-SERCA2a-Thr^484^ axis and Furin contribute more broadly to the pathogenesis of heart failure. As the SERCA2a-Thr^484^Ala mutation blunts the insulin sensitivity of mutant hearts and cardiomyocytes but has no effect on the whole body global insulin response or glucose tolerance, this raises the question of whether different SERCA isoforms are compensatory in other metabolic tissues such as adipose tissues and skeletal muscle and whether this insulin-PKB/AKT-SPEG-SERCA signaling pathway functions similarly in those tissues and organs.
